# Non-destructive determination of Malondialdehyde (MDA) distribution in oilseed rape leaves by laboratory scale NIR hyperspectral imaging

**DOI:** 10.1038/srep35393

**Published:** 2016-10-14

**Authors:** Wenwen Kong, Fei Liu, Chu Zhang, Jianfeng Zhang, Hailin Feng

**Affiliations:** 1 School of Information Engineering, Zhejiang A&F University, Lin’an, Hangzhou, 311300, China; 2 College of Biosystems Engineering and Food Science, Zhejiang University, Hangzhou, 310058, China

## Abstract

The feasibility of hyperspectral imaging with 400–1000 nm was investigated to detect malondialdehyde (MDA) content in oilseed rape leaves under herbicide stress. After comparing the performance of different preprocessing methods, linear and nonlinear calibration models, the optimal prediction performance was achieved by extreme learning machine (ELM) model with only 23 wavelengths selected by competitive adaptive reweighted sampling (CARS), and the result was R_P_ = 0.929 and RMSEP = 2.951. Furthermore, MDA distribution map was successfully achieved by partial least squares (PLS) model with CARS. This study indicated that hyperspectral imaging technology provided a fast and nondestructive solution for MDA content detection in plant leaves.

Plants are exposed to biotic and abiotic stresses in natural environments during their whole life circle. The frequently-occuring abiotic stresses includes extreme temperature, high salinity, excessive light, water deprivation, pollutants such as ozone and herbicides, high concentration of heavy metals, excessive ultra violet radiation and so on. Plants will stimulate the formation of reactive oxygen species (ROS) under abiotic stress, which can harm the production of biomolecules such as lipids, proteins and nucleic acids^1^. Peroxidation of membrane lipid is a major damaging effect of ROS. Usually, membrane lipid peroxidation in plants is detected by measuring malondialdehyde (MDA). MDA is a widely used marker of oxidative lipid injury caused by environmental stress. A number of studies have investigated MDA of plants under different stress conditions. Zhou *et al.*^2^ studied forest trees grown in soil which was exposed to Pb with different levels of water stress, and the results indicated that water stress significantly increased superoxide dismutase (SOD) and peroxidase (POD) activities and MDA content under different Pb concentrations. The data from Jbir-Koubaa^3^ suggested that salinity stress might cause a shock and photo-oxidative stress, which caused MDA accumulation in leaves. Jin *et al.*^4^ studied physiological responses of oilseed rape under herbicide ZJ0273 stress, and the results indicated that MDA contents showed a linear trend with the increasing of ZJ0273. The above studies indicated that lipid peroxidation was a common phenomenon in plants under stress, and MDA could be used as an important indicator of physiological status during plant growth.

Traditional method to detect MDA concentration in plants is thiobarbituric acid (TBA) reactive substances test. High performance liquid chromatography (HPLC) was also employed to determinate MDA in plant tissues^5^. However, the above methods to detect MDA in plants were laborious, lots of chemical regent consumption and requiring complex sample preparation. Our study was investigated to develop a nondestructive and rapid method for MDA analysis using hyperspectral imaging.

Hyperspectral imaging technology is an emerging rapid and nondestructive analytical method widely used in many fields, such as agriculture, food quality and safety assessment, pharmaceutical industries and so on. Hyperspectral imaging can provide spatial and spectral information of each pixel, which can realize the chemical constituent distribution of research objects. EIMasry *et al.*^6^ applied hyperspectral imaging technique to estimate and mapping the water, fat and protein contents in fresh beef. Zou *et al.*^7^ developed a technique for non-destructive chlorophyll estimation and distribution in cucumber leaves using hyperspectral imaging. Higa *et al.*^8^ achieved predictive models and distribution map of water content in golden pothos leaves by hyperspectral imaging. The above studies indicated the feasibility of hyperspectral imaging technology for fast and noninvasive physiological parameter detection in plants.

The main experiment object is oilseed rape (*Brassica napus* L.) which is an important economic crop and widely planted in the world. The objectives were (1) to validate the feasibility of MDA determination using hyperspectral imaging; (2) to compare the performance of different preprocessing methods including moving average smoothing, baseline correction, multiplicative scatter correction, standard normalized variate, de-trending and second derivative, and the performance of different effective wavelength selection methods including weighted regression coefficient, competitive adaptive reweighted sampling, successive projections algorithm, and uninformation variable elimination combined with successive projections algorithm; (3) to develop and compare the linear partial least squares (PLS), nonlinear least squares-support vector machine (LS-SVM) and extreme learning machine (ELM) model; (4) to achieve a distribution map of MDA content in oilseed rape leaves.

## Results

### MDA content and spectral reflectance of oilseed rape leaves

**Figure 1:**
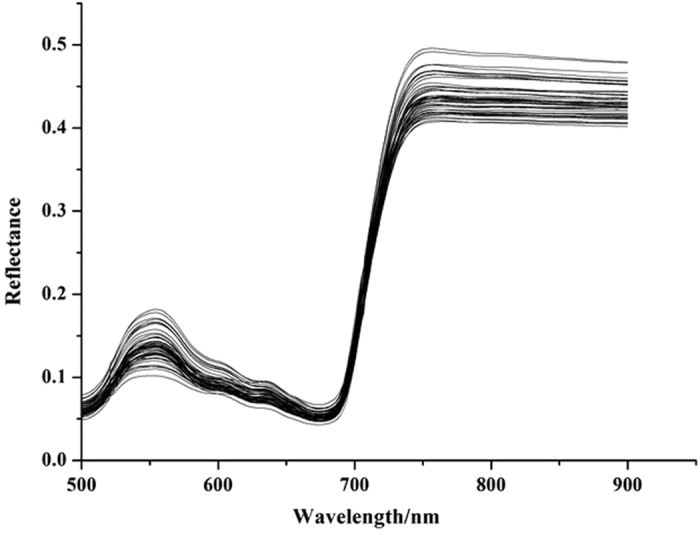
The original Vis/NIR reflectance spectra of oilseed rape leaves under different herbicide stress. The wavelength region was 500–900 nm, and the significant reflectance peak around 550 nm and absorbance peak around 680 nm were caused by chlorophyll.

The spectra of oilseed rape leaves in visible and near-infrared spectral region were shown in Fig. 1. The trends of all samples were quite similar by visual inspection. It was a typical green plant spectral curve with a significant reflectance peak around 550 nm and an absorbance peak around 680 nm caused by chlorophyll. The statistical values of MDA content in oilseed rape leaves were shown in Table 1. In Table 1, a wide range of MDA values were obtained by the stress of two different herbicide varieties and several concentration levels, which would be quite helpful to develop a reliable and robust model.

### Results of full-spectral models

**Figure 2:**
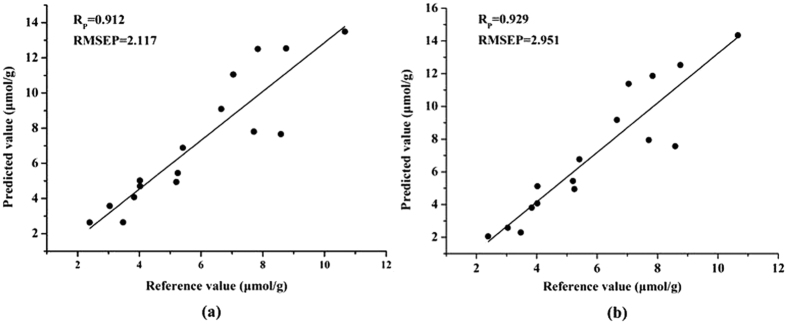
The scatter plots of prediction set by (**a**) full-spectrum PLS model and (**b**) EW-based ELM model. The PLS model applied 500–900 nm as inputs and ELM model applied selected effective wavelengths. The ELM models with R_p_ = 0.929 was better than PLS model with R_p_ = 0.912.

Taking a full-spectrum region of 500–900 nm (316 bands) as input variables, PLS models were built using different preprocessing methods and the results were shown in Table 2. The correlation coefficients of calibration (R_c_) and prediction (R_p_), and root mean square error of prediction (RMSEP) were employed to evaluate the model performance. A good model should have higher R_c_ and R_p_ values, and lower RMSEP values. Comparing the results (seen in Table 2), all PLS models obtained acceptable performance with R_P_ over 0.8. The optimal PLS model achieved R_P_ = 0.912 and RMSEP = 2.117 using moving average smoothing (MAS) and 2-Derivative spectral preprocessing, and the scatter plots of prediction set were shown in Fig. 2.

### Selected effective wavelengths

The full-spectrum region contained 316 bands which might contain high dimensionality and multi-colinearity problems. In order to reduce the input variables and multi-colinearity, 4 effective wavelength (EW) selection strategies were tested, including weighted regression coefficient (WRC), competitive adaptive reweighted sampling (CARS), successive projections algorithm (SPA), and uninformation variable elimination combined with successive projections algorithm (UVE-SPA). The proper preprocessing methods were also taken into consideration for the optimal model performance. The selected wavelengths were shown in Table 3. After EW selection, the input variables were reduced from 316 to less than 23. These selected EWs contained the most important information and were helpful to reduce the computation time, simplify the model and improve the prediction performance.

### Performance of EW-based models

The performance of three regression models was evaluated, including nonlinear PLS model, and nonlinear LS-SVM and ELM models using selected EWs as input variables. The prediction performance was presented in Table 4. Comparing EW-based PLS models (Table 4) and full-spectrum PLS models (Table 2), the EW-based WRC-PLS model and CARS-PLS model showed better results, which indicated that EWs selected by WRC and CARS were effective and contained the most relevant information to represent the full-spectral region. SPA and UVE-SPA were also considered as useful methods to select EWs, since only 4% of the total wavelengths were used and gave similar results compared with full-spectrum models. It was also observed that ELM achieved a better performance than PLS and LS-SVM models. Therefore, ELM was considered as the optimal model for MDA content prediction in oilseed rape leaves. The optimal prediction results using ELM combined with CARS were R_P_ = 0.929 and RMSEP = 2.951, and the scatter plots for these results were shown in Fig. 2. In this study, the least number for EWs was 9 selected by WRC, and the optimal model using these 9 EWs obtained R_P_ = 0.924 and RMSEP = 2.767, which was also better than the full-spectrum PLS models. The above results might give a promising way to develop portable instrument or in-field sensors for oilseed rape growth monitoring.

### Visualization of MDA content in oilseed rape leaves

**Figure 3:**
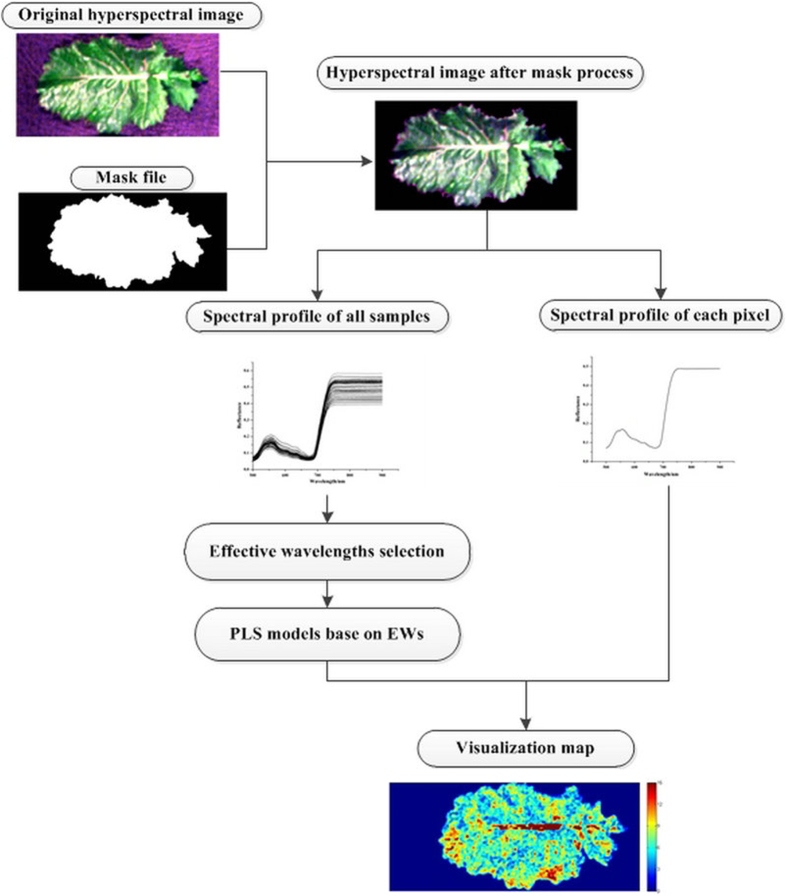
Main steps of visualization including mask of hyperspectral imaging, ROI selection of leaf area without the main leaf vein, effective wavelength-based PLS model development and visualization map. Firstly, the mask file was used to remove the background of original hyperspectral imaging. Secondly, the spectral data were used to select effective wavelengths and develop PLS models, then the pixel information was used as inputs of PLS model, and the predicted value was used to present a visualization map.

**Figure 4:**
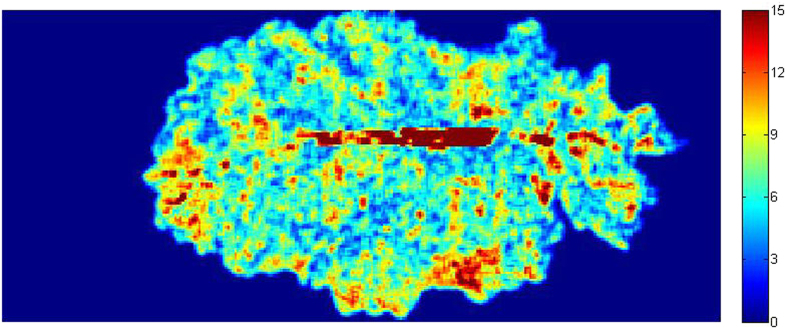
Distribution map of MDA content in oilseed rape leaves built by PLS model. Different colors present different MDA values. The MDA value increased from blue (0) to red (15 μmol/g FW).

Hyperspectral imaging can provide spatial and spectral information of each pixel as mentioned above, so when the spectrum of each pixel was substituted into the regression model, the chemical values of each point could be predicted and then the distribution of MDA of oilseed rape leaves can be achieved^9^,^10^,^11^. Once the PLS model was built, it was applied to predict the MDA content of each pixel within hyperspectral images. The preprocessed hyperspectral image was firstly reduced to multi-spectral image at selected effective wavelengths, then the 3D multi-spectral image was transformed into a 2D data matrix with rows as the coordinates of the pixels and the columns as the reflectance value of the effective wavelengths. The MDA content was calculated by the model, resulting in a one Y column matrix. The Y column was then reshaped to a 2D matrix with the correct pixel coordinates, and a gray-scale image was formed, and then a colorbar with different color was set to the image to show the distribution value of MDA content. The changes of MDA content in a same sample were impossible to be observed by the traditional test methods. The distribution map proved a clear vision of MDA variations in the same leaves, which was a very helpful tool to understand the physiological status of oilseed rape leaves under stress. It might be helpful to detect herbicide stress in an earlier stage before irreversible damages and yield loss occurred. PLS model was a widely used model in visualization because of its simple structure and high computation speed^12^,^13^,^14^. In this study, PLS model and Matlab 2009a were employed to transfer the hyperspectral image into the MDA distribution map. The process of visualization was shown in Fig. 3. Taking one leaf for example, the final MDA content distribution map of oilseed rape leaf was shown in Fig. 4. It was expressed in pseudo color by a linear colorbar with blue indicating low MDA content and red indicating high MDA content. It was noticed that the density of red color in main leaf vein was much higher than other parts. In this study, the visualization of man leaf vein did not indicate high MDA content. The reason was (1) only the leaf area without main leaf vein were used for MDA determination as reference values, which was suggested by agriculture experts; (2) we did not use the main leaf vein area data in the calibration stage. When preceded the hyperspectral image treatment using ENVI software, firstly the region of interest (ROI) was selected manually with a small leaf area, secondly a ROI tool with ‘Grow’ function was applied to select the whole ROI which excluded the main leaf vein automatically, thirdly the selected whole ROI without main leaf vein was applied to develop calibration models; (3) when proceeded the mapping stage, the whole leaf was used and a visualization map was obtained. The hyperspectral image information of main leaf vein area exceeded the calibration range and threshold value, and was marked by red color. In this study, the visualization map of leaf without main leaf vein could be used to indicate the MDA content according the colorbar.

## Discussion

The purpose of this study was to analyze the applicability of hyperspectral imaging technology to detect MDA content in oilseed rape leaves. MDA was an important resistant physiological index of plant under stress. In this study, herbicide was employed as stressors. It was shown in Fig. 2 that the spectral profiles varied from each other. These differences were caused by chemical composition difference. The relationship between MDA and NIR hyperspectra was explored using five spectral preprocessing methods, four effective wavelength selection algorithms and three calibration methods. The performance of preprocessing strategies was assessed by full-spectrum PLS models, then the optimal preprocessing strategy was taken into consideration in EW selection and calibration development stage. Comparing the results between Tables 2 and 4, the optimal prediction performance was achieved using ELM model with 23 wavelengths selected by CARS, and the results were R_P_ = 0.929 and RMSEP = 2.951.

In order to make a comparative study, we performed additional experiments on rice leaves using the same hyperspectral imaging system, and quinclorac herbicide was used as a different stress factor. Two japonica rice (*Oryza sativa* L.) cultivars Zhejiang 88 (ZJ 88) and Xiushui 134 (XS134) were widely planted in southeast China and were selected for this study. The same wavelength range of 500–900 nm was investigated in intensive experimental work. The quinclorac herbicide with the concentration of 0.25 g/L was applied at four-leaf stage. Some of rice samples were treated with salicylic acid (SA) 10 mg/L two days before quinclorac treatment, and these samples were used as control group. MDA measurement was carried out at 10 days after herbicide treatment. A total of 32 samples were prepared, 22 samples were used to develop a calibration model and the other 10 samples were used as prediction set. After a fully comparison similar with oilseed rape leaves, the optimal prediction performance was achieved by ELM mode with 22 wavelengths selected by CARS, and the results was R_P_ = 0.937 and RMSEP = 1.672. PLS model with WRC was employed to generate MDA distribution map of rice leaves. Both the results of models and distribution map produced acceptable results (the detail results and map could be seen in the Supplementary Information file). This additional experiment also indicated that hyperspectral imaging technology could be used to detect MDA in different plants.

Spectral information was widely used for nondestructive testing in many fields, including the application of agriculture^15^,^16^. Hyperspectral imaging captures spatial and spectral information at the same time. Therefore, we could predict the chemical values of each pixel and obtain a chemical distribution visualization map by spectral-based models. The distribution map could not be achieved by imaging or spectroscopic technique alone. Visualization map was an effective tool to study the chemical changes and variations in the same sample. This study confirmed that hyperspectral imaging technology provided an alternative way for a fast and nondestructive detection of MDA in oilseed rape leaves, and the visualization map provided a more direct and understandable way compared with previous determination techniques. However, hyperspectral imaging was still not well developed technology and much more work should be done to meet the requirements of utilization. It was important to develop a robust model with high prediction accuracy and proper methods for assessment of distribution map. Other challenges for utilization might be related to the computation speed, hard ware cost and professional analysis software in the future work.

## Methods

### Sample preparation

A field experiment was conducted at the experimental farm of Zhejiang University, Hangzhou, China. Seeds of oilseed rape (*Brassica napus* cv. ZS758) were provided by College of Agriculture and Biotechnology of Zhejiang University, China. The conventional crop management was used. The rapeseeds were sown in the field of a silt-loam soil with initial 0.18% total nitrogen, 1.75% organic matter and 63 mg/kg soil available phosphorus. Basal fertilizer was applied for this seedling stage experiment. Watering, weeding and other managements were all preceded by farmers according to oilseed rape growth requirements. Various concentrations of benazolin-ethyl [0, 0.25%, 0.50%, 0.75% and 1.00% in volume ratio] and ZJ0273 [0, 100, 200, 500 and 1000 mg/L] were foliar applied at the 5-leaf stage. The benazolin-ethyl was supplied by Anhui Huaxing Chemical Industry Co. Ltd, China. ZJ0273 was supplied by Shanghai Institute of Organic Chemistry, Chinese Academy of Sciences, China. MDA measurement was carried out at 7, 14 and 21 days after herbicide treatment. The fresh leaves were separated and classified as different samples according to the leave position. 20 samples for each herbicide treatment and 9 samples for check test (0 in volume ratio for benazolin-ethyl and 0 mg/L for ZJ0273) were collected for hyperspectral imaging acquisition and MDA measurement. A total of 49 samples were prepared in this study. Each sample was collected from 5 whole oilseed rape plants. In the model development stage, the calibration set (33 samples) was used to develop a calibration model, and the prediction set (16 samples) was applied to assess and evaluate the prediction performance of the developed model. The prediction set didn’t participate in any step of calibration procedure. The samples in three data sets were randomly changed several times to confirm the randomization. No single sample was used in both calibration and prediction sets at the same time.

### NIR hyperspectral imaging acquisition

**Figure 5:**
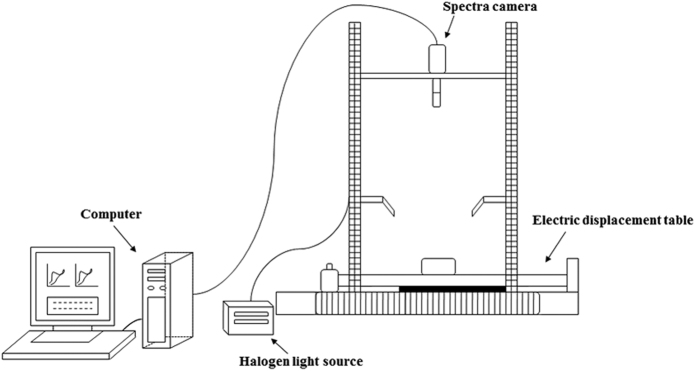
Overview of hyperspectral imaging system including light source, spectra camera, electric displacement table and computer. The electric displacement table can move from right to left with a changeable velocity, the Halogen light source was used to supply desired wavelengths, the spectra camera was used to collect the hyperspectral imaging, and the computer was used to control the system, store and process the data.

A line-scan push-broom hyperspectral imaging system was used in this study. The major elements of this system include light source (2900 Lightsource, Illumination Technologies Inc., USA), spectra camera (ImSpectorV10E, Spectral imaging LTD., Finland), electric displacement table (IRCP0076, Isuzu Optics Crop, Taiwan) and computer for data collection. An overview of the hyperspectral imaging (HSI) system is shown in Fig. 5. The hyperspectral range is from 400 to 1000 nm with a spectral band resolution of 2.8 nm. Leaf samples were placed on the table, two halogen lamps were used as light source and a stepper motor permitted the device to scan samples. The data were recorded in a dark chamber. All images were firstly corrected with a dark and white reference. The dark image was obtained by covering the lens with a cap, and the white image was obtained by white reference board. The corrected reflectance value was calculated as follows:


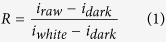


### MDA determination

MDA content in fresh leaves were determined according to Zhou and Leul^17^ with some modification. Fresh leaves (0.3 g) without main leaf vein were homogenized and extracted at 4 °C in 5 ml 0.05 M PBS (pH 7.8). The extract and 2.5 ml thiobarbituric acid were mixed and heated at 100 °C for 15 min, then quickly cooled in an ice bath. After centrifuging at 4800 r/min for 10 min, the absorbance of the supernatant was measured at 450, 532 and 600 nm respectively.

### Spectral preprocessing

Some obvious scattering noises at the beginning and the end of the spectrum were excluded in data processing stage. The remaining 316 bands with spectral range of 500–900 nm were employed in this study. Masking was carried out to isolate samples from the background. All pixels in the fresh leaves without the main leaf vein area were selected as region of interest (ROI), and the average spectra of ROI was calculated as the sample spectra. Preprocessing strategies were adopted to eliminate interfering variability from the spectral information. Five different preprocessing methods were used in this study, including moving average smoothing (MAS), baseline correction, multiplicative scatter correction (MSC), standard normalized variate (SNV), de-trending and second derivative (2-Der). The performance was compared to achieve optimal preprocessing methods.

### Selection of effective wavelengths

A raw hyperspectral image for oilseed rape leaves contained 316 spectral bands, and these contiguous wavelengths might suffer high dimensionality and multi-colinearity problems. Generally, suitable wavelength selection methods could extract the most effective wavelengths (EWs) from hundreds of wavelengths and produce better and simpler models. In this study, four effective wavelength selection strategies were compared, including weighted regression coefficient (WRC), competitive adaptive reweighted sampling (CARS), successive projections algorithm (SPA), and uninformation variable elimination combined with successive projections algorithm (UVE-SPA). The main purpose was to reduce the colinearity problem and reduce the dimensionality of calibration date, and the selected EWs would be more helpful for on-field monitoring sensor development.

### Multivariate analysis

Many effective algorithms were employed to solve multivariate regression problems. These algorithms were classified into two main types, including linear and non-linear regression algorithms. In this study, three multivariate regression algorithms were evaluated, including linear partial least squares (PLS), nonlinear least squares-support vector machine (LS-SVM) and extreme learning machine (ELM). PLS was a popular liner regression algorithm, and widely used in spectral calibration analysis. PLS was performed by creating new variables (principal components or relevant variables) that correspond to the projection of the independent and dependent variables, and then the relationship between new variables and target attributes was built by mathematical method^18^. The details of PLS could be found in the literature^19^. LS-SVM was an extension of standard SVM and a powerful algorithm for solving nonlinear problems. It transformed the quadratic programming problem of a standard SVM demand solution to a linear problem by using the least square value function and equality constraints^20^. The main advantage of LS-SVM was high solution speed and accuracy using small sample set. The theory and more details of LS-SVM could be found in the literatures^21^,^22^. ELM was an emerging learning neural algorithm. The structure of ELM contained one hidden layer and one linear output layer. Comparing learning algorithms with neural networks, ELM could be trained much faster because its input weights were randomly generated and the output weights were analytically computed with a least squares solution^23^. The ELM theory and details were demonstrated in the literature^24^. The correlation coefficient (R) and root mean square error of prediction (RMSEP) were employed to evaluate the quality of developed models, and a better model should have lower RMSEP and high correlation coefficient.

### Employed software

Hyperspectral image acquisition software and matching system were supplied by Isuzu Optics Crop., Taiwan. ENVI 4.7 (ITT Visual Information Solutions, Colorado, USA) was used to extract target information. Unscrambler 10.1 (CAMOAS, Oslo, Norway) and Matlab 2009a (The Math Works, Natick, USA) were used for spectral preprocessing, effective wavelength selection, multivariate analysis and MDA mapping.

## Additional Information

**How to cite this article**: Kong, W. *et al.* Non-destructive determination of Malondialdehyde (MDA) distribution in oilseed rape leaves by laboratory scale NIR hyperspectral imaging. *Sci. Rep.* **6**, 35393; doi: 10.1038/srep35393 (2016).

## Supplementary Material

Supplementary Information

## Figures and Tables

**Table 1:** Statistical values of MDA content in oilseed rape leaves (μmol/g FW).

Data set	Sample No.	Range	Mean	Standard deviation
Calibration	33	2.087–13.141	5.119	2.677
Validation	16	2.390–10.661	5.868	2.392

**Table 2:** Prediction results of MDA by PLS model with different pretreatment.

Pretreatment	R_C_	R_P_	RMSEP
MAS	0.952	0.893	1.764
MAS + Baseline Correction	0.956	0.893	1.863
MAS + MSC	0.950	0.890	1.861
MAS + SNV + De-trending	0.948	0.891	1.810
MAS + 2-der	0.996	0.912	2.117

**Table 3:** Effective wavelengths of MDA prediction selected by different methods.

Pretreatment	Methods	No.	EW/nm
MAS + 2-der	WRC	9	536, 690, 670, 606, 885, 721, 644, 503, 683
	CARS	23	524, 536, 537, 549, 563, 608, 642, 644, 673, 681, 703
			721, 736, 752, 753, 772, 786, 801, 808, 833, 843, 850, 868
	SPA	12	644, 505, 650, 841, 824, 813, 862, 773, 825, 804, 726, 816
	UVE-SPA	13	512, 536, 503, 544, 534, 549, 502, 551, 514, 529, 525
			557, 555

**Table 4:** Prediction results of MDA by different models with EWs.

Pretreatment	EWs selection methods	Models	R_C_	R_P_	RMSEP
MAS + 2-der	WRC	PLS	0.988	0.913	1.955
		LS-SVM	0.992	0.921	1.860
		ELM	0.994	0.924	2.767
	CARS	PLS	0.996	0.919	2.130
		LS-SVM	0.999	0.923	2.092
		ELM	0.998	0.929	2.951
	SPA	PLS	0.993	0.902	2.614
		LS-SVM	0.994	0.906	2.511
		ELM	0.980	0.921	3.246
	UVE-SPA	PLS	0.993	0.910	1.979
		LS-SVM	0.998	0.908	2.226
		ELM	0.982	0.924	2.339

## References

[b1] Gill S. S. & Tuteja N. Reactive oxygen species and antioxidant machinery in abiotic stress tolerance in crop plants. Plant Physiology and Biochemistry 48, 909–930 (2010). 20870416 10.1016/j.plaphy.2010.08.016

[b2] Zhou F. R., Wang J. X. & Yang N. Growth responses, antioxidant enzyme activities and lead accumulation of Sophora japonica and Platycladusorientalis seedlings under Pb and water stress. Plant Growth Regulation 75, 383–389 (2015).

[b3] Jbir-Koubaa R. S. *et al.* Investigation of the response to salinity and to oxidative stress of interspecific potato somatic hybrids grown in a greenhouse. Plant Cell Tissue and Organ Culture 120, 933–947 (2015).

[b4] Jin Z. L. *et al.* Differential morphological and physiological responses of two oilseed Brassica species to a new herbicide ZJ0273 used in rapeseed fields. Pesticide Biochemistry and Physiology 98, 1–8 (2010).

[b5] Davey M. W. *et al.* High-throughput determination of malondialdehyde in plant tissues. Analytical Biochemistry 347, 201–207 (2005). 16289006 10.1016/j.ab.2005.09.041

[b6] EIMasy G., Sun D. W. & Allen P. Chemical-free assessment and mapping of major constituents in beef using hyperspectral imaging. Journal of Food Engineering 117, 235–246 (2013).

[b7] Zou X. B. *et al.* *In vivo* noninvasive detection of chlorophyll distribution in cucumber (Cucumissativus) leaves by indices based on hyperspectral imaging. Analytica Chimica Acta 706, 105–112 (2011). 21995916 10.1016/j.aca.2011.08.026

[b8] Sakura H., Hikaru K. & Satoru T. Mapping of Leaf Water Content Using Near-Infrared Hyperspectral Imaging. Applied Spectroscopy 67, 1302–1307 (2013). 24160882 10.1366/13-07028

[b9] Dai Q. *et al.* Prediction of total volatile basic nitrogen content using wavelet features from visible/near infrared hyperspectral images of prawn (Metapenaeusensis). Food Chemistry 197, 257–265 (2015). 26616948 10.1016/j.foodchem.2015.10.073

[b10] Wang W. *et al.* Feasibility of detecting Aflatoxin B_1_ in single maize kernel using hyperspectral imaging. Journal of Food Engineering 166, 182–192 (2015).

[b11] Burud I. *et al.* Qualitative and quantitative mapping of biochar in a soil profile using hyperspectral imaging. Soil & Tillage Research 155, 523–531 (2016).

[b12] Xie A. G., Sun D. W., Xu Z. Y. & Zhu Z. W. Rapid detection of frozen pork quality without thawing by Vis-NIR hyperspectral imaging technique. Talanta 139, 208–215 (2015). 25882428 10.1016/j.talanta.2015.02.027

[b13] Markus S. & Henning B. Laboratory imaging spectroscopy of a stagnicLuvisol profile – High resolution soil characterisation, classification and mapping of elemental concentrations. Geoderma 195, 122–132 (2013).

[b14] Mostafa K. *et al.* Comparison of Visible–Near infrared and short wave infrared hyperspectral imaging for the evaluation of rainbow trout freshness. Food Research International 56, 25–34 (2014).

[b15] Stubbs T. L., Kennedy A. C. & Fortuna A. Using NIRS to predict fiber and nutrient content of dryland cereal cultivars. Journal of Agricultural and Food Chemistry 58, 398–403 (2010). 19961223 10.1021/jf9025844

[b16] Liu F. *et al.* Determination of acetolactate synthase activity and protein content of oilseed rape (*Brassica napus* L.) leaves using visible/near infrared spectroscopy. Analytical Chimica Acta 629, 56–65 (2008). 10.1016/j.aca.2008.09.027 18940321

[b17] Zhou W. J. & Leul M. Uniconazole-induced alleviation of freezing injury in relation to changes in hormonal balance, enzyme activities and lipid peroxidation in winter rape. Plant Growth Regulation 26, 41–47 (1998).

[b18] Diago M. P. *et al.* Identification of grapevine varieties using leaf spectroscopy and partial least squares. Computers and Electronics in Agriculture 99, 7–13 (2013).

[b19] Wold S., Ruhe A., Wold H. & Dunn W. J. The collinearity problem in linear regression. The Partial Least Squares (PLS) approach to generalized inverses. SIAM Journal on Scientific Computing 5, 735–743 (1984).

[b20] Liu Y. D. & Zhou Y. R. Quantification of the soluble solids content of intact apples by Vis-NIR transmittance spectroscopy and the LS-SVM method. Spectroscopy 28, 32–38 (2013).

[b21] Suykens J. A. K. & Vandewalle J. Least squares support vector machine classifiers. Neural Processing Letters 9, 293–300 (1999).

[b22] Liu F. & Wang L. Comparison of calibrations for the determination of soluble solids content and pH of rice vinegars using visible and short-wave near infrared spectroscopy. Analytica Chimica Acta 610, 196–204 (2008). 18291129 10.1016/j.aca.2008.01.039

[b23] Li W., Chen C., Su H. J. & Du Q. Local binary patterns and extreme learning machine for hyperspectral imagery classification. IEEE Transactions on Geoscience and Remote Sensing 53, 3681–3693 (2015).

[b24] Huang G. B., Zhu Q. Y. & Siew C. K. Extreme learning machine: Theory and applications. Neurocomputing 70, 489–501 (2006).

