# Supplementary material for: Non-destructive determination of Malondialdehyde (MDA) distribution in oilseed rape leaves by laboratory scale NIR hyperspectral imaging
Source: Sci Rep. 2016 Oct 14;6:35393. doi: 10.1038/srep35393 (PMC5064365; doi:10.1038/srep35393)
Supplement: Supplementary Information [file srep35393-s1.doc]

**Non-destructive determination of Malondialdehyde (MDA) distribution in oilseed rape leaves by laboratory scale NIR hyperspectral imaging**

Wenwen Kong^1^, Fei Liu^2^, *, Chu Zhang^2^, Jianfeng Zhang^1^, Hailin Feng^1^

^1^School of information engineering, Zhejiang A&F University, Lin’an, Hangzhou, 311300, China

^2^College of Biosystems Engineering and Food Science, Zhejiang University, Hangzhou, 310038, China

 *Corresponding author: Fei Liu, E-mail: [fliu@zju.edu.cn](mailto:fliu@zju.edu.cn), Tel./Fax.:+86-571-88982825

**Supplementary information:**

**1. The steps for develop visualization map using PLS model.**

When preceded the hyperspectral image treatment using ENVI software, firstly the region of interest (ROI) was selected manually with a small leaf area (seen the following Figure A), secondly a ROI tool with ‘Grow’ function (seen the following Figure B) was applied to select the whole ROI which excluded the main leaf vein automatically (seen the following Figure C), thirdly the selected whole ROI without main leaf vein was applied to develop calibration models.

| 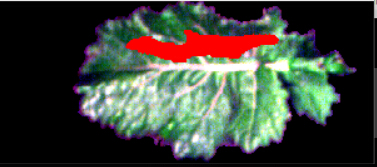 | 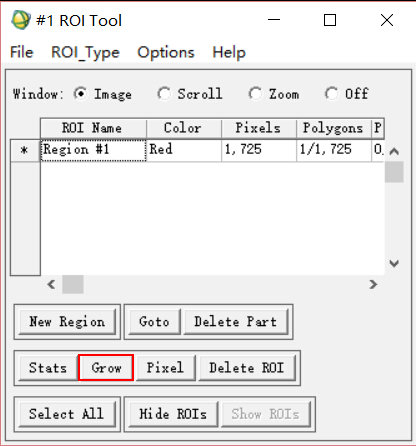 |
| --- | --- |
| **Figure A** |
| 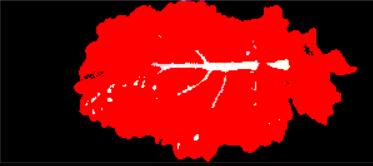 |
| **Figure B** | **Figure C** |

**2. The prediction results of MDA prediction in rice leaves (Table A) and the MDA distribution map by WRC-PLS model (Figure D).**

**Table A** Prediction results of MDA in rice leaves by different models with moving average smoothing (MAS) pretreatment and EWs

| EWs selection methods and wavelengths | Models | R_C_ | R_P_ | RMSEP |
| --- | --- | --- | --- | --- |
| WRC (710, 895, 500, 554, 672 and 752nm) | PLS | 0.905 | 0.891 | 3.139 |
|  | LS-SVM | 0.981 | 0.873 | 3.380 |
|  | ELM | 0.913 | 0.874 | 2.214 |
| CARS (534, 554, 558, 580, 581, 605, 640, 642, 654, 667, 687, 700, 714, 735, 753, 801, 814, 820, 840, 841, 865 and 900 nm) | PLS | 0.995 | 0.884 | 3.078 |
|  | LS-SVM | 0.978 | 0.552 | 6.843 |
|  | ELM | 0.981 | 0.937 | 1.672 |
| SPA (558, 900, 895, 548, 764, 648, 667, 506, 693, 742, 702, 831, 683, 726, 884, 774, 515, 500, 714 and 590 nm) | PLS | 0.908 | 0.882 | 3.203 |
|  | LS-SVM | 0.987 | 0.857 | 3.198 |
|  | ELM | 0.916 | 0.918 | 1.427 |
| UVE-SPA (667, 679, 746, 719 and 778 nm) | PLS | 0.953 | 0.838 | 3.513 |
|  | LS-SVM | 0.989 | 0.828 | 3.666 |
|  | ELM | 0.978 | 0.898 | 2.160 |


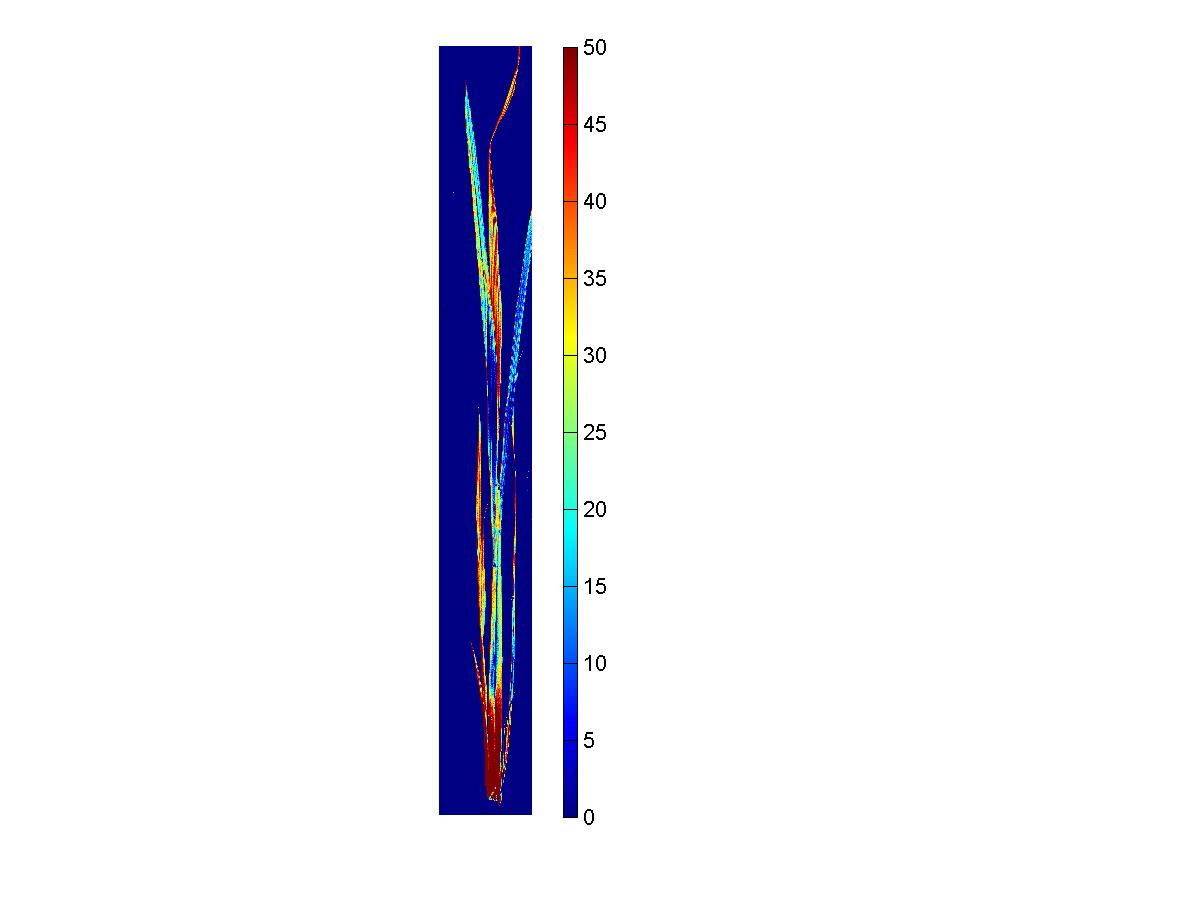


**Figure D**
